# Study on Effect of Nano-CaCO_3_ on Properties of Phosphorus Building Gypsum

**DOI:** 10.3390/ma16093354

**Published:** 2023-04-25

**Authors:** Yi Zhang, Zhong Tao, Lei Wu, Zhiqi Zhang, Zhiman Zhao

**Affiliations:** 1 Faculty of Civil Engineering and Mechanics, Kunming University of Science and Technology, Kunming 650500, China; 20201110003@stu.kust.edu.cn (Y.Z.); wulei0324@163.com (L.W.);; 2 Yunnan Earthquake Engineering Research Institute, Kunming 650500, China; 3 Yunnan Ningchuang Environmental Technology Co., Ltd., Anning 650300, China

**Keywords:** phosphorus building gypsum, nano-CaCO_3_, mechanical properties

## Abstract

Phosphogypsum is an industrial by-product from the wet preparation of phosphoric acid. Phosphorus building gypsum (PBG) can be obtained from phosphogypsum after high-thermal dehydration. Improving the mechanical properties of PBG is of great significance to extending its application range. In this paper, PBG was modified by adding nano-CaCO_3_. Specifically, this study, conducted on 0.25–2% nano-CaCO_3_-doped PBG, tested effects on the fluidity, setting time, absolute dry flexural strength, absolute dry compressive strength, water absorption and softening coefficient of PBG, followed by its microscopic analysis with SEM and XRD. The experimental results showed that, with an increase in nano-CaCO_3_ content, the fluidity and setting time of PBG-based mixes were decreased. When the content was 2%, the fluidity was 120 mm, which was 33% lower than that of the blank group; the initial setting time was 485 s, which was 38% lower than that in the blank group; the final setting time was 1321 s, which was reduced by 29%. Nano-CaCO_3_ evidently improved the absolute dry flexural strength, absolute dry compressive strength, water absorption and softening coefficient of PBG to a certain extent. When the content was 1%, the strengthening effect reached the optimum, with the absolute dry flexural strength and absolute dry compressive strength being increased to 8.1 MPa and 20.5 MPa, respectively, which were 50% and 24% higher than those of the blank group; when the content was 1.5%, the water absorption was 0.22, which was 33% lower than that of the blank group; when the content approached 0.75%, the softening coefficient reached the peak of 0.63, which was 66% higher than that of the blank group. Doping with nano-CaCO_3_ could significantly improve the performance of PBG, which provides a new scheme for its modification.

## 1. Introduction

Phosphogypsum is an industrial by-product from the wet preparation of phosphoric acid. Its major component is CaSO_4_·2H_2_O, which is usually gray-white or gray-black. About 4~5 t of phosphogypsum is produced per 1 t of phosphoric acid. At present, the annual discharge of phosphogypsum in China is about 30 million t [1,2,3], which is one of the largest solid wastes from the chemical industry. Phosphorus building gypsum (PBG) can be obtained from phosphogypsum after high-thermal dehydration. The major component is CaSO_4_·1/2H_2_O. When PBG is used as a building material, it exhibits low strength and poor water resistance due to a large number of pores, which makes it difficult to be more widely applied [4,5,6,7].

To improve various properties of PBG, other materials are usually introduced as reinforcement. Wu [8,9,10] studied the effects of short-cut basalt fiber, glass fiber and polypropylene fiber on the properties of PBG and obtained the best mixing ratio of these different fibers. Liang [11] explored the influences of ordinary silicate cement on the mechanical properties of PBG, with the results showing that the addition of this cement could effectively promote the late strength of PBG. Ma [12] investigated the doping effects of the amount of recycled brick powders and the type of activators on the compressive strength and water resistance of PBG, with the results exhibiting that the introduction of brick powders could raise the compressive strength of PBG. Li [13] researched the impacts of doping limes and cements on the strength and water resistance of PBG-based cementitious materials and found these properties of the modified materials were both significantly increased in the later stages. Ji [14] applied multi-wall carbon nanotube materials to modify the PBG to make its internal structure more dense and its mechanical properties stronger. Zhao [15] adopted fly ashes and silica fumes to modify PBG-based mortar, which also improved its performance.

Nano-materials are ultra-fine, with particle sizes less than 100 nm [16,17]. This type of nano-admixture has already been broadly applied to, and has significantly improved the various properties of, building materials [18,19,20,21,22]. Given that the ultra-refinement of nano-CaCO_3_ particles changes the crystal structure and surface electronic structure, many characteristics have been generated [23,24]. The research of Kawashima [25] showed that, after doping with nano-CaCO_3_, the initial setting and final setting times of the cement were both shortened, while its hydration rate was significantly improved. Camiletti [26] studied the effect of nano-CaCO_3_ on the early properties of ultra-high performance concrete, with the test results exhibiting that nano-CaCO_3_ could improve the early mechanical properties of cement-based materials. The research of Liu [27] presented that the addition of nano-CaCO_3_ could promote both the compressive strength and flexural strength of cement-based materials at the optimal content of 1%. Detwiler [28] found that for the hydration product, C-S-H gel, of cements, the action of nano-CaCO_3_ crystal nuclei, accelerated its formation rate on the particle surface. The research results of Qian [29] demonstrated that nano-CaCO_3_ was able to fill the pores of cement-based materials, making the concrete structure more compact, improving its mechanical properties.

Despite these study results, there remains a lack of research on the modification of PBG by nano-CaCO_3_. In this study, nano-CaCO_3_ was added into PBG as a reinforcing material to study its influence on fluidity, setting time, absolute dry flexural strength, absolute dry compressive strength, water absorption and softening coefficient of PBG, explore its optimal content and analyze its influence mechanism. The properties of PBG can be improved effectively by adding nano-CaCO_3_ into PBG, so that PBG can be widely used. At the same time, nano-CaCO_3_ is a type of environmental protection material. The combination of nano-CaCO_3_ and PBG and their application in the building materials industry not only meet the requirements of green building materials, but also achieves waste utilization. Therefore, using nano-CaCO_3_ to modify PBG is a problem worth studying.

## 2. Materials and Methods

### 2.1. Raw Materials

Phosphogypsum: light-yellow powder from a phosphogypsum yard of Yunnan Yuntianhua Co., Ltd. (Kunming, China). Its chemical composition is shown in Table 1.

This phosphogypsum was washed with water, neutralized with lime and then dried at 130 °C for 6 h to obtain PBG.

**Figure 1:**
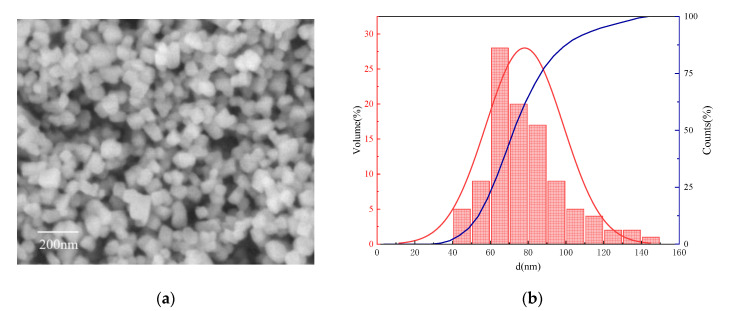
(**a**) SEM photo of nano-CaCO3; (**b**) Particle size distribution.

Nano-CaCO_3_: produced by Jiangxi Bairui Calcium Carbonate Co., Ltd. (Yichun, China). The SEM and particle size distribution are shown in Figure 1 and the technology parameters are shown in Table 2.

### 2.2. Experimental Design

Different contents of nano-CaCO_3_ were added into PBG according to Table 3, stirred and dispersed evenly, then added to water, mixed evenly and poured into a 40 mm × 40 mm × 160 mm mold; mixtures then underwent vibration for 30 s on the vibration table.

### 2.3. Experimental Methods

#### 2.3.1. Fluidity and Setting Time

For each mixture proportion, the fluidity of specimens was measured using the gypsum consistency testing meter and the initial and final setting times of specimens were measured using the Vicat apparatus, according to the Chinese national standard “Gypsum plasters Determination of physical properties of pure paste” (GB/T17669.4-1999) [30].

#### 2.3.2. Absolute Dry Flexural Strength and Absolute Dry Compressive Strength

For each mixture proportion, three specimens were molded for the tests. Specimens were demolded after curing at 25 °C, 50 ± 5% RH for 24 h and cured at the same environment for 7 days. Then, all the specimens were dried at the temperature of 45 °C in an electric thermostatic drying oven until the weight was constant. The absolute dry compressive strength and absolute dry flexural strength of specimens were tested in accordance with the Chinese national standard “Determination of Mechanical Properties of Building Plaster” (GB/T 17669.3-1999) [31].

#### 2.3.3. Water Absorption and Softening Coefficient

Three specimens were immersed in water for 24 h and the saturated specimens were prepared by wiping off the water on their surfaces with towels. The masses of the specimens were measured by an electronic balance with 0.01 g accuracy. The breaking load of the specimens, F, was measured according to the standard GB/T 17669.3-1999. The water absorption was calculated by Formula (1) and the softening coefficient was calculated by Formula (2):

W = (m_1_ − m_0_)/m_0_, (1)

K = F_2_/F_1_, (2)

where W is the water absorption, m_0_ is the dry mass of the sample and m_1_ is the mass of the sample saturated in water.

In addition, K is the softening coefficient, F_1_ is the breaking load of the dry sample and F_2_ is the breaking load of the saturated sample.

#### 2.3.4. Microscopic Morphology of Gypsum Particles

A small number of samples were taken from the middle of the broken block, from which the micromorphology was observed under a scanning electron microscope after vacuum metal spraying.

## 3. Results

### 3.1. Effect of Nano-CaCO_3_ on PBG Fluidity

Table 4 shows the influence of different contents of Nano-CaCO_3_ on properties of PBG.

**Figure 2:**
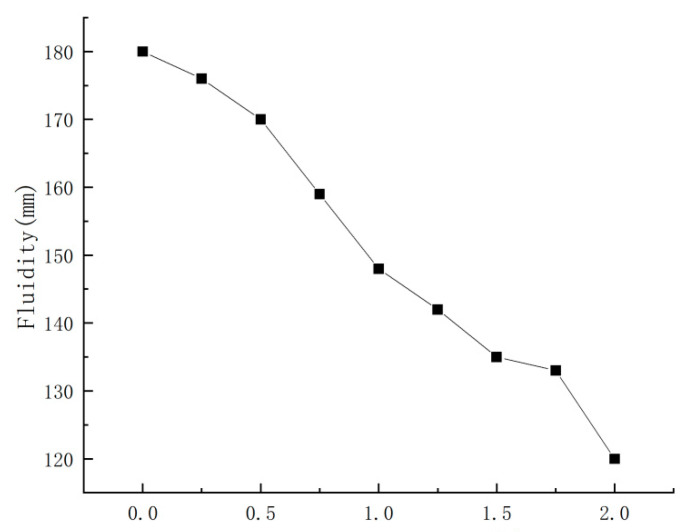
Effects of nano-CaCO_3_ on the fluidity of PBG.

Firstly, the water requirement was adjusted to make the fluidity of PBG in the blank group reach 180 mm, then nano-CaCO_3_ was added according to the mixing proportions in Table 3 to compare the effects of different contents of nano-CaCO_3_ on the PBG fluidity. The comparison results were shown in Figure 2.

It could be seen from Figure 2 that, with the increase in nano-CaCO_3_ content, the fluidity of PBG decreased continuously. When the content of nano-CaCO_3_ was 2%, the fluidity of PBG was reduced to 120 mm as the minimum, which was 33% lower than that of the blank group.

### 3.2. Effect of Nano-CaCO_3_ on Setting Time of PBG

**Figure 3:**
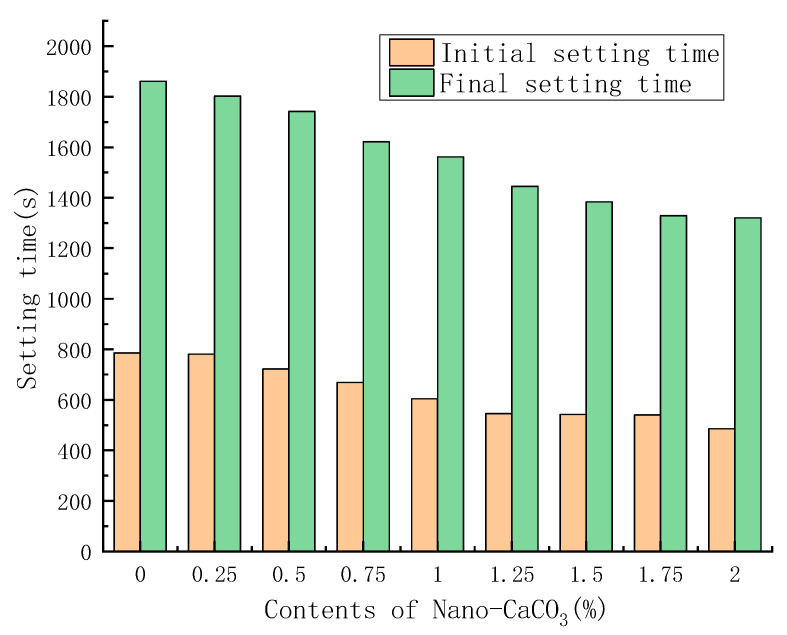
Effects of nano-CaCO_3_ on setting times of PBG.

With the water requirement of standard consistency of PBG being unchanged, nano-CaCO_3_ was added according to the mixing proportions in Table 3 to compare the effects of different contents of nano-CaCO_3_ on the initial and final setting times of PBG. The comparison results were shown in Figure 3.

It could be seen from Figure 3 that, with the increase in nano-CaCO_3_ content, the initial and final setting times of PBG were both continuously shortened. When the content of nano-CaCO_3_ reached 2%, the initial setting time of PBG fell to 485 s as the minimum, which was 38% lower than the 786 s of the blank group; also, the final setting time dropped to 1321 s as the minimum, which was 29% lower than the 1861 s of the blank group.

### 3.3. Effect of Nano-CaCO_3_ on Absolute Dry Flexural Strength of PBG

**Figure 4:**
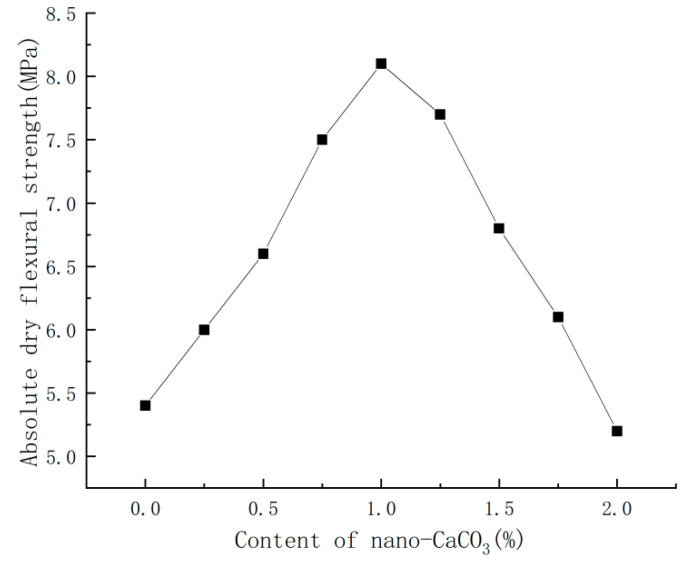
Effects of nano-CaCO_3_ on absolute dry flexural strength of PBG.

With the water requirement of standard consistency being unchanged, the content of nano-CaCO_3_ was varied to compare the corresponding changes in absolute dry flexural strength of PBG. The comparison results were shown in Figure 4.

It could be seen from Figure 4 that, with the increase in nano-CaCO_3_ content, the absolute dry flexural strength of PBG showed a trend of first increasing then decreasing. When the content of nano-CaCO_3_ was 1%, the absolute dry flexural strength of PBG reached 8.1 MPa as the maximum, which was 50% higher than that of the blank group; when the content of nano-CaCO_3_ exceeded 1%, the absolute dry flexural strength of PBG began to decrease. When the content of nano-CaCO_3_ was 2%, the absolute dry flexural strength of PBG was 5.2 MPa, which was 4% lower than that of the blank group.

### 3.4. Effect of Nano-CaCO_3_ on Absolute Dry Compressive Strength of PBG

**Figure 5:**
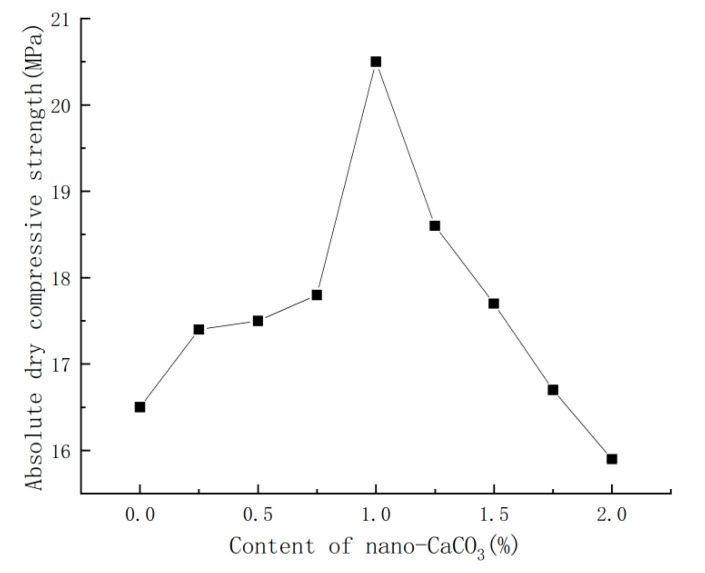
Effects of nano-CaCO_3_ on absolute dry compressive strength of PBG.

With the water requirement of standard consistency being unchanged, the content of nano-CaCO_3_ was varied to compare the corresponding changes in absolute dry compressive strength of PBG. The comparison results were shown in Figure 5.

It could be seen from Figure 5 that, with the increase in nano-CaCO_3_ content, the absolute dry compressive strength of PBG showed a trend of first increasing then decreasing. When the content of nano-CaCO_3_ was 1%, the absolute dry compressive strength of PBG rose to 20.5 MPa as the maximum, which was 24% higher than that of the blank group; when the content of nano-CaCO_3_ exceeded 1%, the absolute dry compressive strength of PBG started to decrease. When the content of nano-CaCO_3_ reached 2%, the absolute dry compressive strength of PBG dropped to 15.9 MPa, which was 4% lower than that of the blank group.

### 3.5. Effect of Nano-CaCO_3_ on Water Absorption of PBG

**Figure 6:**
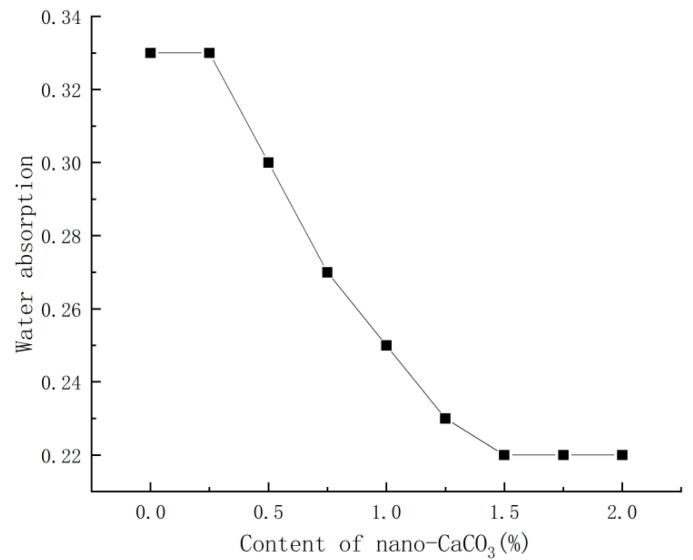
Effects of nano-CaCO_3_ on water absorption of PBG.

With the water requirement of standard consistency being unchanged, the content of nano-CaCO_3_ was varied to compare the corresponding changes in PBG’s water absorption. The comparison results were shown in Figure 6.

It could be seen from Figure 6 that, with the increase in nano-CaCO_3_ content, the water absorption of PBG first decreased then stabilized. When the content of nano-CaCO_3_ reached 1.5%, the water absorption of PBG was 0.22, which was 33% lower than that of the blank group; when the content of nano-CaCO_3_ exceeded 1.5%, the water absorption of PBG gradually stabilized.

### 3.6. Effect of Nano-CaCO_3_ on Softening Coefficient of PBG

**Figure 7:**
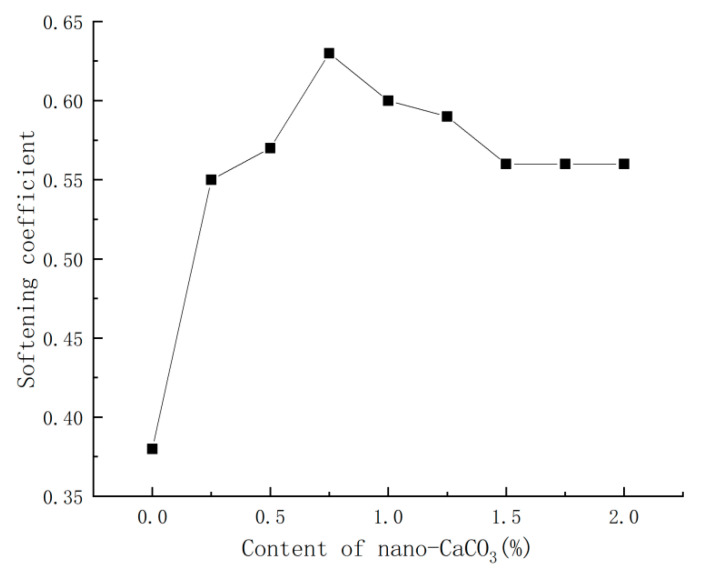
Effects of nano-CaCO_3_ on softening coefficient of PBG.

With the water requirement remaining unchanged, the content of nano-CaCO_3_ was varied for comparing the corresponding changes of PBG’s softening coefficient. The comparison results were shown in Figure 7.

It could be seen from Figure 7 that, with the increase in nano-CaCO_3_ content, the softening coefficient of PBG first increased then decreased, before tending to stabilize. When the content of nano-CaCO_3_ was 0.75%, the softening coefficient of PBG rose to 0.63 as the maximum, which was 66% higher than that of the blank group; when the content of nano-CaCO_3_ exceeded 0.75%, the softening coefficient of PBG began to decrease; when the content of nano-CaCO_3_ reached 1.5%, the softening coefficient of PBG gradually stabilized. At that moment, the softening coefficient remained at 0.56, which was 47% higher than that of the blank group.

### 3.7. Microanalysis

**Figure 8:**
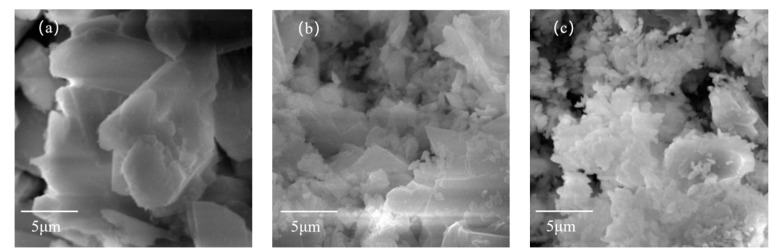
(**a**) SEM photo of PBG; (**b**) SEM photo of PBG mixed with 1% nano-CaCO_3_; (**c**) SEM photo of PBG mixed with 2% nano-CaCO_3._

The microstructures of PBG prototypes and mixtures with nano-CaCO_3_ were separately observed by scanning electron microscopy (SEM). The results were shown in Figure 8.

It could be seen from Figure 8a,b that, after adding nano-CaCO_3_, a lot of granular nano-CaCO_3_ with tiny particle size appeared on the surface of PBG. This nano-CaCO_3_ wrapped PBG crystals and filled the gaps between them, which reduced the total porosity and exerted the significant effect of micro-aggregation. However, when the added content became too high, CaCO_3_ would become unevenly dispersed. It could be seen from Figure 8c that the unevenly dispersed CaCO_3_ still wrapped the crystals of PBG. Although the porosity was further reduced, these unevenly dispersed parts generated stress concentration, which imposed an adverse impact on the strength of PBG.

### 3.8. Composition Analysis of Hydration Products

**Figure 9:**
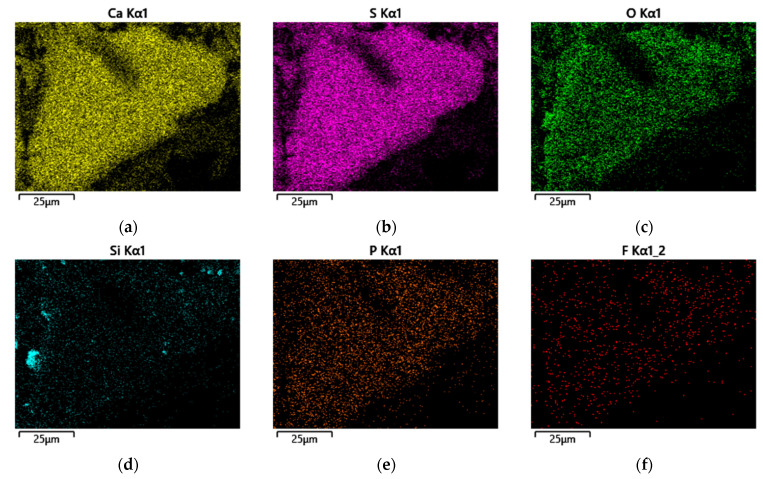
EDS element mapping results of PBG mixed with 1% nano-CaCO_3._ (**a**) Ca mapping results; (**b**) S mapping results; (**c**) O mapping results; (**d**) Si mapping results; (**e**) P mapping results; (**f**) F mapping results.

**Figure 10:**
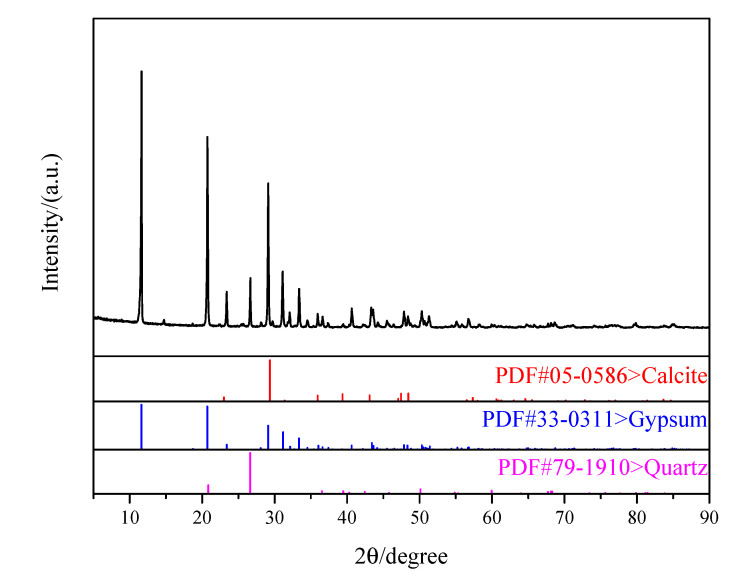
Composition of hydration products.

The EDS element mapping images and XRD patterns were characterized and shown in Figure 9 and Figure 10, respectively.

It could be seen from EDS and element mapping images (Figure 9a–f) that the main elements in the selected area were Ca (20.3%), S (18.1%) and O (60.5%), together with trace amounts of P (0.8%) and Si (0.3%). The P element in the sample was uniformly dispersed, while the Si element appeared as concentrated spots in very limited numbers, representing the quartz crystals detected by XRD (Figure 10). There were no P-containing crystals detected by XRD, due to its high dispersion (Figure 9e). This also could be seen from Figure 10 that, after adding nano-CaCO_3_ into PBG, these two had not yet directly reacted with each other and the main product from the hydration of PBG was still CaSO_4_·2H_2_O [32,33,34].

## 4. Discussion

It can be seen from the above test data that, after nano-CaCO_3_ was added into PBG, its fluidity and water absorption were both reduced, while its absolute dry flexural strength, absolute dry compressive strength and softening coefficient were all improved, with the absolute dry flexural strength seeing the largest improvement. The main reasons are as follows:

Filling effect: Since the average particle size of nano-CaCO_3_ is about 100 nm, which is far below that of PBG, a proper amount of nano-CaCO_3_ can fill both the micropores of PBG and the internal pores of PBG’s hydration product. Simultaneously, it can also improve the particle gradation and change the pore structure of PBG’s hydration product by reducing macropores, increasing micropores and lowering the total porosity. The ability to fill the space between PBG particles is greatly promoted, the PBG’s total porosity is reduced and the PBG’s structure is denser, which improves the PBG’s absolute dry flexural strength, absolute dry compressive strength and softening coefficient in all and reduces its water absorption as well.

Nucleation effect: The main component of PBG is CaSO_4_·1/2H_2_O and its hydration reaction is mainly with water to produce CaSO_4_·H_2_O. When PBG starts hydration, the hydrolysis of CaSO_4_·1/2H_2_O releases a large amount of Ca^2+^. After nano-CaCO_3_ is added to PBG, it does not directly participate in the hydration reaction, but, compared with ordinary CaCO_3_, the surface activity of nano-CaCO_3_ is relatively high, which, therefore, adsorbs the Ca^2+^ released by hydration. This causes the CaSO_4_·2H_2_O around nano-CaCO_3_ to nucleate in advance, which leads to the decrease in Ca^2+^ concentration in the solution and the increase in Ca^2+^ migration from CaSO_4_·1/2H_2_O, thus accelerating the hydration efficiency of PBG. Based on the original structure, a new one is formed around nano-CaCO_3_ as the crystal nucleus, which reduces both the internal surface area and porosity of PBG, increases its compactness and improves its various properties.

Pinning effect: Meanwhile, the existence of nano-CaCO_3_ particles in PBG also generates a “pinning effect”. This can be seen from Figure 8b, that some nano-CaCO_3_ particles are embedded into the gaps of PBG’s hydration product. When the hydration product is compressed to generate microcracks inside, their expansion is hindered by nano-CaCO_3_ particles and their energy is consumed, which limits the crack propagation and improves various properties of PBG [35].

With the increase in nano-CaCO_3_ content, the absolute dry flexural strength and absolute dry compressive strength of PBG both decrease. This is because the surface energy of nano-CaCO_3_ is relatively large and, when the content becomes too high, nano-CaCO_3_ agglomerates instead of evenly dispersing within PBG. While PBG is subjected to external force, these agglomerated nano-CaCO_3_ particles have stress concentration, which affects both the absolute dry flexural strength and absolute dry compressive strength of PBG [36].

Moreover, the specific surface area of nano-CaCO_3_ is extremely large. After mixing with water, this surface adsorbs a large amount of water, which reduces the water required to participate in the hydration reaction of PBG. Therefore, with the increase in nano-CaCO_3_ content, PBG’s fluidity and setting time both decrease. If excessive nano-CaCO_3_ is added, PBG’s strength is also affected [37]. However, when the softening coefficient is measured, part of the PBG without hydration continues to hydrate, resulting in the supplement to the wet strength of PBG and less reduction compared to the absolute dry strength, so the softening coefficient is improved [38].

## 5. Potential Applications and Prospects

The composition of the PBG is relatively complicated, with substantial impurities, which have a negative impact on its performance. According to the results of this paper, it can be seen that, after doping with nano-CaCO_3_, various properties of the PBG have all been improved, which would meet the application requirements for building gypsum. Compared to other materials, nano-CaCO_3_ presents certain advantages, as listed in Table 5. Compared to other preparation methods, the method adopted in this paper also has advantages, in that there is no waste generated in the preparation process and it poses not only the merits of simplicity, environmental protection and low cost, but also the certain practical significance in production.

## 6. Conclusions

1. Nano-CaCO_3_ exerted a significant effect on the physical properties of PBG’s paste with the increase in nano-CaCO_3_ content: the fluidity and setting time of PBG were both decreased. Among them, the fluidity decreased by 33%, the initial setting time decreased by 38% and the final setting time decreased by 29%.
2. Nano-CaCO_3_ also presented a significant impact on the mechanical properties of PBG: with the increase in nano-CaCO_3_ content, both the absolute dry flexural strength and absolute dry compressive strength of PBG first increased then decreased. When the content of nano-CaCO_3_ was 1%, the absolute dry flexural strength of PBG increased by 50% and the absolute dry compressive strength increased by 24%. When the content of nano-CaCO_3_ reached a certain level, it imposed a negative impact on the mechanical properties of PBG.
3. Nano-CaCO_3_ also had a significant influence on other properties of PBG: with the increase in nano-CaCO_3_ content, the water absorption of PBG first decreased then stabilized gradually, decreasing by 33%, while the softening coefficient first increased, then decreased and finally tended to stabilize, with a maximum increase of 66%.
4. After nano-CaCO_3_ was added into PBG, it could fill the voids within the hardened body and improve PBG’s pore structure. Meanwhile, based on the original structure, it could form a new one around nano-CaCO_3_ as the crystal nucleus, which reduced both the internal surface area and porosity of PBG, increased PBG’s compactness and improved PBG’s various properties.

## Figures and Tables

**Table 1:** Chemical content analysis of phosphogypsum.

Component	SiO_2_	Al_2_O_3_	Fe_2_O_3_	MnO	MgO	CaO	K_2_O	P_2_O_5_	SO_3_	Organism
**Content (%)**	14.52	1.66	0.15	0.005	0.17	31.94	0.22	0.94	45.38	0.25

**Table 2:** Technology parameters of nano-CaCO_3._

Appearance	CaCO_3_ Content (%)	Specific Surface Area (m^2^/g)	Particle Size Distribution			pH
			D10 (nm)	D50 (nm)	D90 (nm)	
White powder	98	22	54.65	74.66	100.73	9.05

**Table 3:** Contents of nano-CaCO_3._

No.	1	2	3	4	5	6	7	8	9
Contents of Nano-CaCO_3_(%)	0	0.25	0.5	0.75	1	1.25	1.5	1.75	2

**Table 4:** Effects of different contents of nano-CaCO_3_ on properties of PBG.

No.	Fluidity (mm)	Initial Setting Time (s)	Final Setting Time (s)	Absolute Dry Flexural Strength (MPa)	Absolute Dry Compressive Strength (MPa)	Water Absorption (%)	Softening Coefficient (%)
1	180	786	1861	5.4	16.5	0.33	0.38
2	176	781	1803	6	17.4	0.33	0.55
3	170	722	1742	6.6	17.5	0.3	0.57
4	159	669	1622	7.5	17.8	0.27	0.63
5	148	604	1562	8.1	20.5	0.25	0.6
6	142	545	1445	7.7	18.6	0.23	0.59
7	135	542	1384	6.8	17.7	0.22	0.56
8	133	540	1329	6.1	16.7	0.22	0.56
9	120	485	1321	5.2	15.9	0.22	0.56

**Table 5:** Comparing effects of nano-CaCO_3_ doping and other treatment methods.

Performance Index	Here	9	10	11	14
Materials	Nano-CaCO_3_	Short-cut polypropylene fiber	Chopped basalt fiber	Ordinary Portland cement	Multi-walled carbon nanotubes
Content (%)	1	1.5	1.6	10	0.03
Fluidity (mm)	148	170	60	-	-
Initial setting time (s)	604	-	610	480	-
Final setting time (s)	1562	-	1530	1200	-
Absolute dry flexural strength (MPa)	8.1	6.46	-	5.1	4.94
Absolute dry compressive strength (MPa)	20.5	17.63	-	16.05	17.46
Softening coefficient	0.6	-	-	0.42	-

## Data Availability

The data supporting this study’s findings are available on request from the corresponding author.
